# 1,3,8-Triazaspiro[4.5]decane Derivatives Inhibit Permeability Transition Pores through a F_O_-ATP Synthase c Subunit Glu^119^-Independent Mechanism That Prevents Oligomycin A-Related Side Effects

**DOI:** 10.3390/ijms24076191

**Published:** 2023-03-24

**Authors:** Gaia Pedriali, Daniela Ramaccini, Esmaa Bouhamida, Alessio Branchini, Giulia Turrin, Elisabetta Tonet, Antonella Scala, Simone Patergnani, Mirko Pinotti, Claudio Trapella, Carlotta Giorgi, Elena Tremoli, Gianluca Campo, Giampaolo Morciano, Paolo Pinton

**Affiliations:** 1 Maria Cecilia Hospital, GVM Care and Research, 48033 Cotignola, Italymrcgpl@unife.it (G.M.); 2 Department of Life Sciences and Biotechnology, University of Ferrara, 44121 Ferrara, Italy; 3 Department of Chemical, Pharmaceutical and Agricultural Sciences, University of Ferrara, 44121 Ferrara, Italy; 4 Cardiology Unit, Azienda Ospedaliero Universitaria di Ferrara, 44124 Cona, Italy; 5 Department of Medical Sciences, University of Ferrara, 44121 Ferrara, Italy

**Keywords:** permeability transition pore, Oligomycin A, c subunit of F_O_-ATP synthase, small molecules, cardioprotection

## Abstract

Permeability transition pore (PTP) molecular composition and activity modulation have been a matter of research for several years, especially due to their importance in ischemia reperfusion injury (IRI). Notably, c subunit of ATP synthase (Csub) has been identified as one of the PTP-forming proteins and as a target for cardioprotection. Oligomycin A is a well-known Csub interactor that has been chemically modified in-depth for proposed new pharmacological approaches against cardiac reperfusion injury. Indeed, by taking advantage of its scaffold and through focused chemical improvements, innovative Csub-dependent PTP inhibitors (1,3,8-Triazaspiro[4.5]decane) have been synthetized in the past. Interestingly, four critical amino acids have been found to be involved in Oligomycin A-Csub binding in yeast. However, their position on the human sequence is unknown, as is their function in PTP inhibition. The aims of this study are to (i) identify for the first time the topologically equivalent residues in the human Csub sequence; (ii) provide their in vitro validation in Oligomycin A-mediated PTP inhibition and (iii) understand their relevance in the binding of 1,3,8-Triazaspiro[4.5]decane small molecules, as Oligomycin A derivatives, in order to provide insights into Csub interactions. Notably, in this study we demonstrated that 1,3,8-Triazaspiro[4.5]decane derivatives inhibit permeability transition pores through a F_O_-ATP synthase c subunit Glu^119^-independent mechanism that prevents Oligomycin A-related side effects.

## 1. Introduction

The mitochondrial permeability transition (mPT) is an altered permeabilization of the inner mitochondrial membrane (IMM) to low-molecular weight solutes, which leads to mitochondrial swelling, failure of mitochondrial functions and cell death [1,2]. High intracellular calcium (Ca^2+^) concentrations, oxidative stress and dyshomeostasis of phosphates, adenine nucleotides and many other substances in the mitochondrial matrix [3] contribute to this pathological condition. The mPT, an evolutionarily-conserved event from yeast to mammals, is caused by the opening of multiple channels in the IMM, namely permeability transition pores (PTP), and also involves the cooperation of the outer mitochondrial membrane (OMM) [4], as well as additional modulatory proteins [5]. Although PTP flickering is recognized to be important in physiology [6,7,8], PTP opening is currently considered a cellular catastrophe representing one critical pathway of cell death in multiple pathologies.

The most studied phenomenon in which PTP opening triggers extensive cell death and tissue function loss is ischemia reperfusion injury (IRI) [9,10,11,12]. In ischemic environments, the PTP is blocked due to the high proton concentration or by cellular acidification due to glycolytic pathways being activated in the absence of oxygen [13]. Otherwise, during reperfusion, PTP opening is prompted by generation of reactive oxygen species (ROS), pH recovery and mitochondrial Ca^2+^ accumulation [12,13].

Several efforts to understand this channel have found many similarities between the regulation of the PTP opening process and some ATP synthase catalytic peculiarities [14,15,16,17]. In 2013, these functional similarities were confirmed by identification of an overlapping structural composition of the two machineries. Indeed, the c subunit of ATP synthase (Csub) has been identified as one of the proteins forming the pore of the PTP [18,19,20], and the dimers of ATP synthase, under some pathological conditions, might disassemble into monomers facilitating the formation of the pores [21].

Notably, Csub expression has been identified as strongly correlated to several end-points of reperfusion damage in patients affected by ST-segment elevation myocardial infarction (STEMI) [22]. In addition, mutations in the genes encoding Csub have been found to be associated with worse clinical outcomes [23], and the absence of Csub protects cells from PTP opening and cell death [24].

The Csub protein has been proposed as a target for new pharmacological approaches against IRI [25,26,27]. In particular, one of the known Csub interactors named Oligomycin A [28,29,30] has been taken into consideration for several studies to develop new Csub-dependent PTP inhibitors. This resulted in a de novo synthesis of small-molecule inhibitors of Csub through multiple modifications of the Oligomycin A chemical structure. In detail, these compounds have been (i) synthetized starting from the 1,7-dioxaspiro[5.5]-undecane moiety of Oligomycin A, considered to be essential in ensuring Csub binding, and (ii) shown to be able to improve PTP inhibition with beneficial effects on the cardiac performance after IRI, but without known side effects that hamper the use of this macrolide for therapeutic purposes [25].

Although, there is experimental evidence from multiple studies on binding of these small-molecules (named as PP11 and compound 10) with the target protein, the evidence was indirect [25,26]. To better define how and to what extent the Oligomycin A-derivatives are able to interact with the Csub protein from the PTP inhibition point of view, we focused on the evidence obtained by the high-resolution crystal structure of the Oligomycin A—Csub complex, especially on the amino acids identified as critical for their binding in yeast [31]. In this study, we identified for the first time the corresponding residues in human Csub. By taking advantage of site-directed mutagenesis and alanine scanning of the human *ATP5G1* sequence, we constructed and tested several Csub variants to evaluate PTP activity modulation by Oligomycin A and its derivatives based on the 1,3,8-Triazaspiro[4.5]decane scaffold (hereafter named as PP11 and C.10).

Unlike Oligomycin A, we provided experimental evidence that PP11 and C.10 do not mediate their PTP inhibitory effect through involvement of the Glu^119^ residue in Csub. These insights provide one direct proof of the binding between the 1,3,8-Triazaspiro[4.5]decane derivatives and Csub. Interestingly, this peculiarity in the ligand–protein interaction might explain their outstanding properties as cardioprotective agents.

## 2. Results

### 2.1. Identification of Human Csub Residues Crucial for Oligomycin A Binding

**Figure 1:**
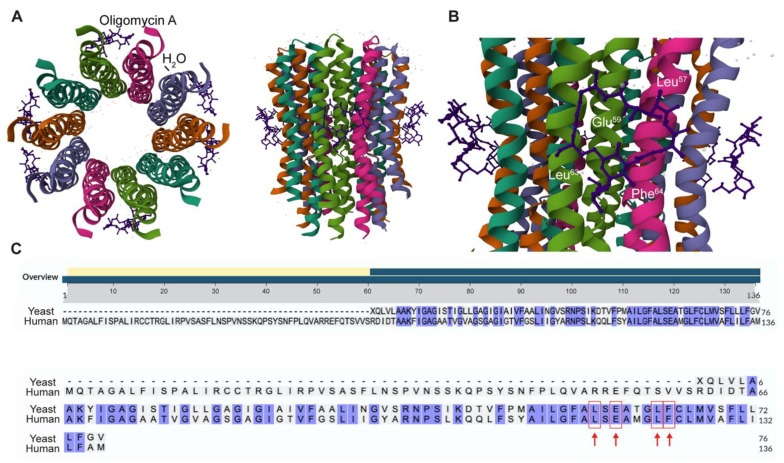
Identification of conserved amino acids involved in Oligomycin A—Csub binding in human sequence. (**A**) High resolution crystal structure (left, top view; right, side view) of Csub binding site in yeast (PDB: 4F4S) and Oligomycin A labeled. Molecules of Oligomycin A are depicted in purple, H_2_O molecules in orange, and Csub chains are colored with the “Chain Id” mode. (**B**) View of the Oligomycin A binding site on yeast Csub. The residues involved in the binding (Leu^57^, Glu^59^, Leu^63^, Phe^64^) are indicated in white. (**C**) Alignment of yeast Csub protein sequence with the human counterpart. All conserved amino acids are highlighted in blue. Red arrows indicate amino acids topologically equivalent to Leu^117^, Glu^119^, Leu^123^ and Phe^124^ in human Csub.

In 2012, the establishment of a high-resolution crystal structure of the complex between Oligomycin A and yeast Csub protein (PDB: 4F4S, [31]) allowed the identification of the amino acids critical for Oligomycin A binding (Figure 1A). Among these amino acids, Leu^57^, Leu^63^ and Phe^64^ were found to have a great impact on direct interactions with Oligomycin A (Figure 1B), while Glu^59^ is involved in indirect but essential binding. Indeed, Glu^59^ binds the macrolide through a hydrogen bond and to a water molecule that also entails binding with the carbonyl group of Leu^57^ [32,33] (Figure 1B). Moreover, these residues, when mutated, conferred resistance to the antibiotic function of Oligomycin A in yeast [31].

The aminoacidic sites participating in interactions with Oligomycin A on the human Csub sequence are currently unknown. It is suggested that the amino acid residues forming the Oligomycin A binding site are 100% conserved between human and yeast [31]. However, yeast and human Csub sequences display a low similarity degree and a different total length (yeast Csub, 76 aa; human Csub, 136 aa). Thus, the identification of the relative position in human Csub of the conserved Leu^57^, Glu^59^, Leu^63^ and Phe^64^ is relevant for structural comparisons.

To this aim, yeast (PDB: 3UD0, residues 1 to 76) and human Csub (NP_005166.1) protein sequences were aligned by the BLAST algorithm from NCBI [34]. Analysis of the alignment (Figure 1C) revealed a 63% degree of similarity, with the key residues in yeast (Leu^57^, Glu^59^, Leu^63^ and Phe^64^) identified as Leu^117^, Glu^119^, Leu^123^ and Phe^124^ in the human sequence.

### 2.2. In Vitro Validation of the Amino Acids Involved in Oligomycin A—Csub Binding in Oligomycin A-Mediated PTP Inhibition

Besides being a strong F_O_-ATP synthase interactor, Oligomycin A is a known PTP inhibitor [35]. To assess the importance of Oligomycin A binding with Csub in PTP inhibition, we took advantage of an alanine scanning approach to generate variants bearing single (ATP5G1^L117A^, ATP5G1^E119A^, ATP5G1^L123A^, ATP5G1^F124A^) or multiple (ATP5G1^4ALA^) substitutions, which are critical for the establishment of this binding.

First, we evaluated the expression and proper localization of protein variants in human cardiac fibroblasts (HCF) cells, which allowed us to investigate PTP inhibition in a cardiac environment [36,37], but with the minimal contribution of endogenous Csub expression. Indeed, being fibroblasts a non-contractile cell type, thus requiring less ATP production compared with cardiomyocytes, they express lower levels of Csub (Figure S1D). Moreover, they also express lower levels of Csub compared with other cell lines mimicking the cardiac tissue (Figure S1A).

**Figure 2:**
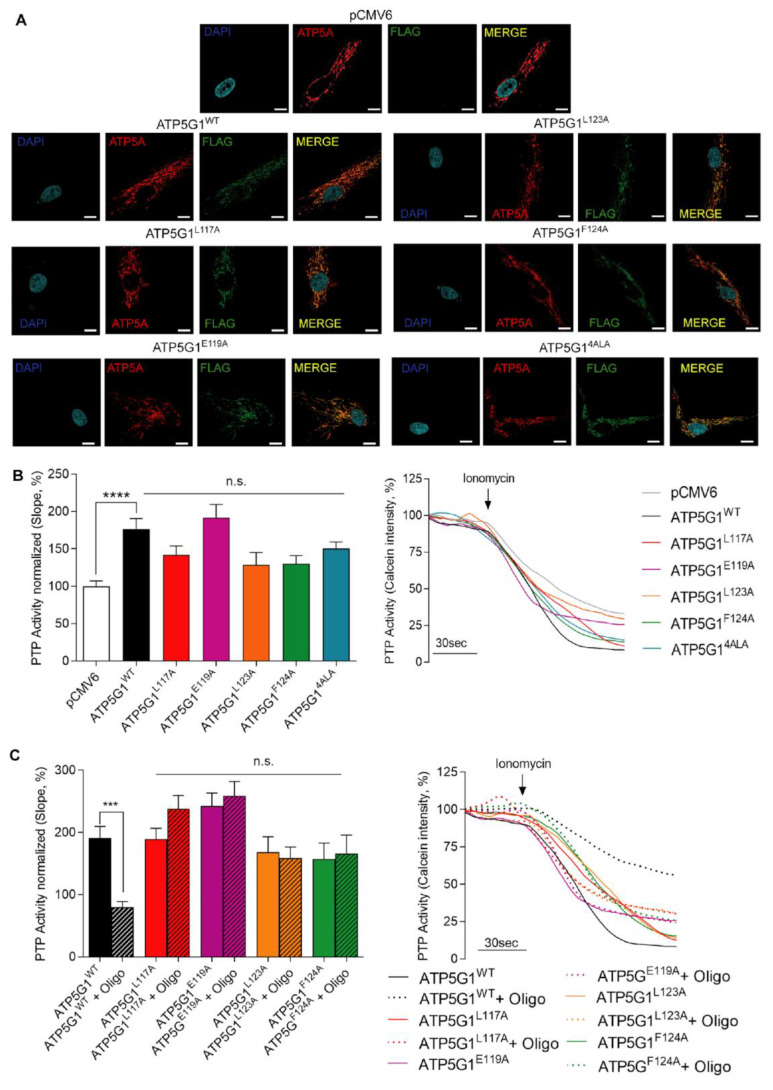
Functional analysis of Oligomycin A-Csub binding in PTP inhibition in primary human cardiac fibroblasts. (**A**) Immunofluorescence assay for mitochondrial localization of mutants: FLAG-tag (green), ATP5A as mitochondrial marker (red), and nuclei (blue, DAPI). Scale bar in white is 10 μm. (**B**) PTP activity measurement (left, analysis of PTP activity; right, representative kinetics) with mutants expressed in HCF and compared with pCMV6 and ATP5G1^WT^. (**C**) PTP activity measurement (left, analysis of PTP activity; right, representative kinetics) with mutants expressed in HCF with or without Oligomycin A pre-treatment (10 μM, 30 min). Graphs show mean ± SEM. Statistical differences were analyzed by one-way ANOVA. ***, *p* < 0.001; ****, *p* < 0.0001, n.s. not statistically significant.

Figure 2A shows immunofluorescence of HCF transfected with the empty vector (pCMV6), the wild-type protein (ATP5G1^WT^) or the ATP5G1^L117A^, ATP5G1^E119A^, ATP5G1^L123A^, ATP5G1^F124A^ and ATP5G1^4ALA^ variants. To confirm their localization at the mitochondrial level, we detected the signal of the mitochondrial marker ATP5A (in red) and the signal expressed by the FLAG-tag (in green), which is present in all plasmids with exception of pCMV6. These data show that all the single mutants are expressed in HCF cells and localized at the mitochondrial level. In addition, the overexpression of FLAG-tagged proteins in HCF cells transfected with all the plasmids was confirmed by Western blotting analysis (Figure S1B).

Then, in each of these experimental conditions, we evaluated PTP opening in living HCF to understand whether mutant overexpression can differently impact PTP activity when compared with ATP5G1^WT^ after 1 μM Ionomycin administration (Figure 2B). The calcein–cobalt quenching assay detected an increased opening of the PTP when ATP5G1^WT^ is overexpressed and compared with pCMV6 (*p* < 0.0001). Moreover, the overexpression of each mutant, including ATP5G1^4ALA^, impacts on PTP opening in a manner comparable with ATP5G1^WT^, with no significant difference among conditions. These findings agree with previous observations on increased PTP opening following ATP5G1^WT^ overexpression, and also suggest that each mutation does not impact on the function of the wild-type form in terms of PTP activity (Figure 2B).

To understand whether Oligomycin A retains its inhibitory activity against PTP when Csub is mutated in the crucial binding pocket, the calcein–cobalt quenching assay was performed in HCF expressing ATP5G1^WT^, ATP5G1^L117A^, ATP5G1^E119A^, ATP5G1^L123A^ and ATP5G1^F124A^. Experimental conditions were subdivided in either vehicle or 30 min pre-treatment with 10 μM Oligomycin A, and the kinetics of the PTP were evaluated. First, we observed that Oligomycin A pre-treatment is able to inhibit PTP opening both in the basal condition (*p* < 0.0001, Figure S1C) and upon ATP5G1^WT^ overexpression (*p* < 0.001, Figure 2C). Interestingly, Oligomycin A failed to exert PTP inhibition when each Csub mutant is expressed. Indeed, we found no statistical differences between PTP activity in cells overexpressing mutants with or without Oligomycin A pre-treatment. Altogether, these findings confirm the key role of these amino acids in Csub-dependent PTP inhibition exerted by the macrolide. In addition, as shown in Supplementary S1, in our conditions, Oligomycin A acute treatment does not affect cell viability nor intracellular ATP levels (Figure S1D,F), but a 24 h treatment has detrimental effects on these parameters, leading to a decrease in cell viability as well as intracellular ATP levels of about 40% (Figure S1E,G). On the other hand, 1,3,8-Triazaspiro[4.5]decane-based small-molecules used in acute or long (24 h) treatment did not display an impact on cell viability nor intracellular ATP levels, confirming a safer effect of these compounds compared with Oligomycin A.

### 2.3. Leu^117^ and Leu^123^ Residues Are Essential for the Binding of PP11 to Csub

**Figure 3:**
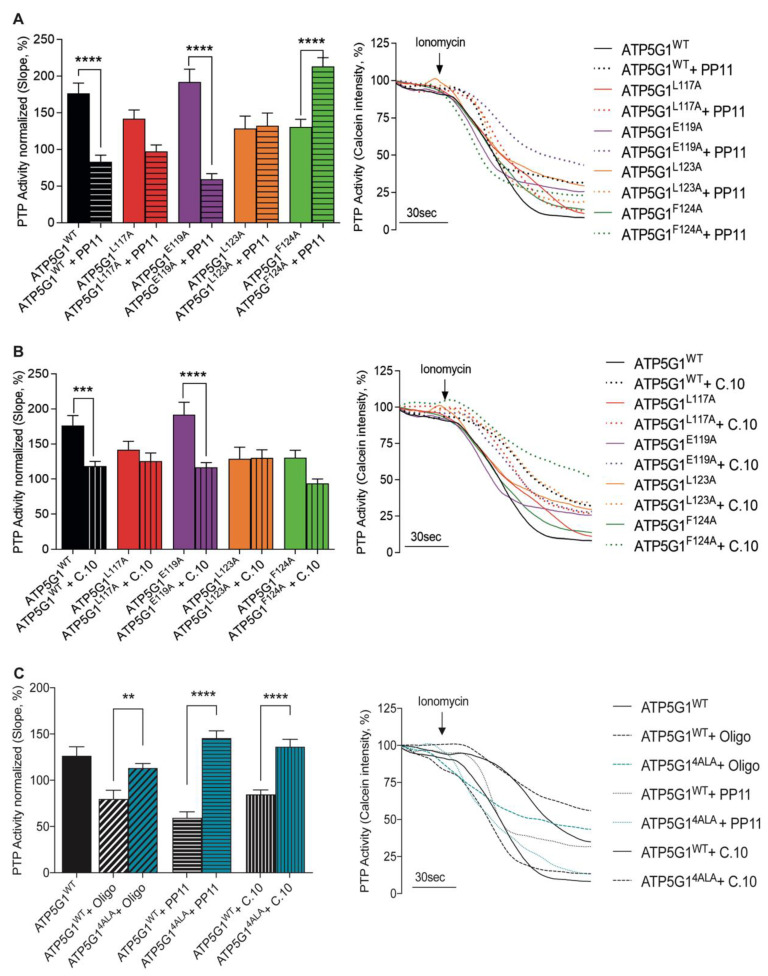
Differential binding of small-molecule inhibitors to Csub variants with mutated residues. (**A**) PTP activity measurement in cells overexpressing Csub variants with PP11 pre-treatment (5 μM, 15 min). On the left, PTP activity; on the right, representative traces of PTP activity. (**B**) PTP activity measurement under conditions of mutants’ overexpression with C.10 pre-treatment (5 μM, 15 min). On the left, PTP activity; on the right, representative traces of PTP activity. (**C**) PTP activity measurement under conditions of ATP5G1^4ALA^ mutant overexpression pre-treated with Oligomycin A, PP11 and C.10 and compared with ATP5G1^WT^. On the left, PTP activity; on the right, representative traces of PTP activity. Graphs show mean ± SEM. Statistical differences were analyzed using one-way ANOVA: **** *p* < 0.0001, *** *p* < 0.001, ** *p* < 0.01.

To determine whether 1,3,8-Triazaspiro[4.5]decane-based small molecules, as Oligomycin A derivatives, inhibit PTP opening due to the presence of the same Oligomycin A chemical core, we first investigated the functional impact of PP11 in cells expressing each mutant. Thus, HCF cells were transfected with ATP5G1^WT^, ATP5G1^L117A^, ATP5G1^E119A^, ATP5G1^L123A^ or ATP5G1^F124A^ constructs and pre-treated either with the vehicle or with 5 μM of the PP11 compound. Through the calcein–cobalt quenching assay, we first confirmed that PP11 inhibits PTP opening in basal conditions (*p* < 0.01, Figure S1H) and after ATP5G1^WT^ overexpression (Figure 3A). Interestingly, when ATP5G1^L117A^ and ATP5G1^L123A^ are the main isoforms expressed in cells, PP11 failed to inhibit PTP opening, suggesting that these amino acids, in their conserved form, are essential for the binding of PP11 and for PTP inhibition. These data agree with those obtained with Oligomycin A. Despite not being statistically significant, we found a decreasing trend in PTP activity after the use of PP11 in cells expressing ATP5G1^L117A^, indicating that the inhibitory effect of this compound was not completely suppressed, and that this amino acid might be less important for Csub binding compared with Leu^123^ (Figure 3A). It is interesting that, different from Oligomycin A, PP11 in HCF expressing ATP5G1^E119A^ continues to exert PTP inhibition. This finding suggests that Glu^119^ is not essential for PP11 binding to Csub and PTP inhibition.

The different data obtained with PP11 molecule pre-treatment suggest that the aromatic moiety in position 1 of the spirocyclic structure might interact with Leu^123^, and that the toluyl portion of the sulphonyl amide in position 8 might interact with Leu^117^. Furthermore, this arrangement would permit the sulfoxide moiety to disrupt the binding of water molecules to Glu^119^, avoiding the blockage of this amino acid that plays a key role in proton translocation, and in turn in ATP synthase activity. This evidence may also explain the fact that, as previously proven by us, treatment with Oligomycin A appears to irreversibly block ATP synthase activity and is, therefore, toxic to cells, unlike our newly designed compounds.

In contrast, when ATP5G1^F124A^ was expressed, PP11 pre-treatment triggered an abrupt increase in PTP opening, suggesting an effect of the expression of this variant only in the presence of the compound (Figure 3A). The possible binding between the hydrogen of the lactame ring in position 3 with the py elecrons Phe^124^ would stabilize the conformational structure of the 1,3,8-Triazaspiro[4.5]decane scaffold. However, once Phe is replaced by Ala, this interaction would be lost, and the entire structure would probably change its shape, causing the activation of PTP.

### 2.4. Leu^117^, Leu^123^ and Phe^124^ Are Essential for the Binding of Compound 10 to Csub

In a similar way, the behavior of C.10 was evaluated in Figure 3B. First, we confirmed that 5 μM C.10 pre-treatment in HCF inhibited PTP opening both in resting conditions, as expected (*p* < 0.0001, Figure S1I), and in cells overexpressing ATP5G1^WT^ (*p* < 0.001, Figure 3B). Cells overexpressing ATP5G1^L117A^, ATP5G1^L123A^ and ATP5G1^F124A^ were not susceptible to the inhibition exerted by C.10, suggesting a key role of these amino acids within the C.10-Csub binding complex in PTP modulation. Nevertheless, C.10 pre-treatment on the ATP5G1^F124A^ variant induced a decrease in PTP activity by 36% compared with the untreated condition. Even if this effect was not statistically significant, we might envision that Phe^124^ is less essential than ATP5G1^L117A^ and ATP5G1^L123A^.

Moreover, unlike Oligomycin A but in line with the PP11 results, pre-treatment with C.10 in cells expressing ATP5G1^E119A^ as the main isoform significantly inhibited PTP opening when compared with untreated cells (*p* < 0.0001). This finding suggests that Glu^119^ is not involved in C.10 binding as well as PP11 (Figure 3B).

In support of these data, in a condition in which all four single residues were mutated and localized to mitochondria (Figure 2A), Oligomycin A, C.10 and PP11 pre-treatment was inefficient in the inhibition of PTP (Figure 3C).

## 3. Discussion

The complex interaction between F_O_-ATP synthase and Oligomycin A has attracted considerable interest in multiple fields of research worldwide, namely as an antiproliferative agent in cancer [38,39,40], as a potent antimicrobial in infectious diseases [41], in the investigation of mitochondrial features, in metabolomics studies, and lately as a PTP inhibitor to counteract cell death in cardiovascular diseases [28,29,30]. Thus, the scaffold of Oligomycin A, and how it interacts with the target, is of great interest in pharmacology [41,42]. However, because of its side effects on healthy cells, the use in therapy in its native form has been always postponed.

There are about ten different conserved amino acids involved in the binding between Csub and the macrolide [31]. Among these, three are shown to have key roles in direct interactions established with Oligomycin A, namely, Leu^57^, Leu^63^ and Phe^64^, as well as Glu^59^, which would form an indirect but essential binding through a hydrogen bond with a water molecule acting as a bridge [33]. Notably, when mutated, they conferred resistance against Oligomycin A action.

For these reasons, and given our interest in human cardiac diseases, we chose to introduce substitutions to alanine in residues corresponding to those (Leu^117^, Glu^119^, Leu^123^ and Phe^124^) of the human Csub sequence to understand their involvement in Oligomycin A-dependent PTP inhibition.

In this context, overexpression of the single ATP5G1^L117A^, ATP5G1^E119A^, ATP5G1^L123A^ or ATP5G1^F124A^ mutants in HCF cells allowed us to monitor a clear functional effect on PTP activity, which was not different from ATP5G1^WT^-induced PTP sensibilization, but crucial for Oligomycin A binding. Indeed, Oligomycin A was able to inhibit PTP opening in cells overexpressing ATP5G1^WT^, but it loses the modulatory effect when each site becomes mutated individually or concurrently. This is a key finding further supporting the key role of Csub in the structure of the PTP.

A second investigation aimed to provide more direct evidence for the suggested binding between new cardioprotective small-molecule PTP inhibitors and Csub [25,26]. In 2018, we conceived and synthesized several classes of PTP inhibitors by taking advantage of the minimal chemical structure of Oligomycin A required for Csub binding. This constituted another important point for the rationale underlying Csub mutagenesis. The small molecules were further modified in order to develop new therapeutic strategies against reperfusion damage, targeting Csub without Oligomycin A side effects such as ATP depletion [25]. We showed the exclusive localization of PP11 and C.10 at a mitochondrial level or at the putative site of action, the thermal stabilization of Csub protein at increasing concentrations of the inhibitors, and their great potential in PTP inhibition and recovery from reperfusion damage [25]. Experiments shown in Figure S1D–G on cell viability and intracellular ATP levels further confirmed our previously published data, demonstrating that these small-molecule derivatives are optimal cardioprotective agents compared with Oligomycin A.

In this study we provided experimental evidence for a behavior of both PP11 and C.10 comparable with that of Oligomycin A which mainly involves three out of four Csub amino acids (Leu^117^, Leu^123^ and Phe^124^), even if the last one is involved to a lesser extent. This finding was expected as these compounds are derivatives of Oligomycin A. However, we were also expecting differences that could have contributed to explaining the absence of appreciable toxic effects of PP11 and C.10 on main mitochondrial and cellular parameters when compared with Oligomycin A [25]. The data reported here point toward a key role of the Csub glutamic acid at position 119, which is not essential for PTP inhibition by PP11 and C.10. Indeed, when mutated, both derivatives inhibit PTP opening through the putative binding with the other essential residues. This observation has significant implications as the PTP inhibition mediated by 1,3,8-Triazaspiro[4.5]decane does not involve the key Glu^119^ residue, which is postulated to participate in proton movement during ATP synthesis, thus preventing the side effects known to be associated with Oligomycin A.

## 4. Materials and Methods

### 4.1. Cell Culture

Human cardiac fibroblasts (HCF) were provided by Innoprot (P10452, Derio, Bezkaia, Spain) and used according to the manufacturer’s instructions. They were cultured in Fibroblast Medium-2 containing fetal bovine serum, fibroblast growth supplement-2, penicillin and streptomycin (Innoprot, P60108-2). All the experiments described in previous sections were performed with the same culture passage (p3-p8). 

### 4.2. Transfection

HCF were transfected with Lipofectamine™ LTX Reagent with PLUS™ Reagent (Thermo Fisher Scientific, Walthan, MA, USA, A12621) according to the manufacturer’s instructions.

Expression vectors were created by site-directed mutagenesis [43] of the human ATP5G1 coding sequence (NM_005175.2 reference sequence) and cloned into the pCMV6-Entry plasmid using the QuickChange II Site-Directed Mutagenesis Kit (Agilent Technologies, Santa Clara, CA, USA). The forward oligonucleotides CTTGGCTTTGCC*GCG*TCTGAGGC CATG (ATP5G1^L117A^), CTTTGCCCTGTCT*GCG*GCCATGGGGCTTTTC (ATP5G1^E119A^), GAGGCCATGGGG*GCT*TTCTGTTTGATG (ATP5G1^L123A^), and GCCATGGGGCTT*GCC*T GTTTGATGGTC (ATP5G1^F119A^) were used to introduce substitutions to alanine and obtain single or double mutants. The modified nucleotides (underlined) and missense triplets (italics) are indicated. Reverse oligonucleotides were complementary to the forward ones. All the plasmids were validated by sequencing.

### 4.3. Immunofluorescence

HCF were transfected with the empty vector (pCMV6), wild-type Csub (ATP5G1^WT^) or the Csub mutants according to the previous protocol. Then, cells were fixed with 4% paraformaldehyde (PFA) in phosphate-buffered saline (PBS) for 15 min at room temperature and permeabilized with 0.1% Triton in PBS for 15 min. After blocking with 2% bovine serum albumin (BSA) in PBS-Triton for 1 h, the cells were incubated with primary antibody at appropriate dilutions for 1 h at 4 °C. The primary antibodies used were anti-ATP5A antibody [15H4C4] (ab14748, Abcam, Cambridge, UK) for mitochondria and ANTI-Flag^®^ (F7425, Merck, Burlington, MA, USA) for c subunit mutants. After washing 2 times with PBS-Triton, the cells were stained with corresponding secondary antibody in BSA 2% under dark conditions. The secondary antibody used were Goat anti-Mouse IgG (H + L) Highly Cross-Adsorbed Secondary Antibody, Alexa Fluor 647 (Thermo Fisher Scientific, A-21236) and Goat anti-Rabbit IgG (H + L) Cross-Adsorbed Secondary Antibody, Alexa Fluor 488 (Thermo Fisher Scientific, A-11008). The cells were again washed 3 times with PBS-Triton before been mounted with the mounting buffer with 4,6-diamidino-2-phenylindole (DAPI) (DUO82040, Sigma-Aldrich, St. Louis, MO, USA).

High-resolution images of the cells were recorded using a FV3000 Olympus confocal laser scanning microscope with a 60× objective lens (Plan-Fluor 60×/Oil, Zeiss, Oberkochen, Germany, image size: 1024 × 1024). The acquired images were then analyzed by using open-source software ImageJ 2.1.0/1.53c https://imagej.nih.gov/ij (accessed on 1 January 2020).

### 4.4. Western Blot

For immunoblotting, the cells were lysed in RIPA Lysis and Extraction Buffer (Thermo Fisher Scientific, 89900), quantified by the Lowry method and then 10 µg of proteins were loaded on a 4–20% precast gel. After electrophoretic separation, proteins were transferred onto nitrocellulose membranes that were incubated overnight with primary antibodies including Anti-β-Actin (1:5000, Sigma-Aldrich, A1978), ANTI-Flag^®^ (F7425, Merck), ANTI-GAPDH (1:5000, 2118L, Cell signaling, Denver, MA, USA) and ANTI-Csub (1:1000, ab181243, Abcam). The revelation was assessed by specific HRP-labelled secondary antibodies, followed by detection by chemiluminescence using ChemiDoc™ Touch Gel Imaging System. The Western blots shown in figures are representative of at least three different independent experiments.

Cardiac cell lines lysates: Human Aortic Endothelial Cells (HAEC) were provided by Thermo-Fisher (C0065C) and cultured according to the manufacturer’s instructions; AC16 Human Cardiomyocyte Cell Line was provided by Merck (SCC109) and cultured according to the manifacturer’s instructions; Human Cardiac Myocytes (HCM) were provided by Promocell (FB60C12810 Heidelberg, Germany) and cultured according to the manufacturer’s instructions.

### 4.5. Calcein–Cobalt Assay

For all the experiments of mPTP activity, HCF were co-transfected with the empty vector (pCMV6), wild-type (ATP5G1^WT^) or mutated (ATP5G1^L117A^, ATP5G1^E119A^, ATP5G1^L123A^, ATP5G1^F119A^, ATP5G1^4ALA^) Csub variants, according to the previous protocol, and histone H2B-RFP vector, in a 1:3 ratio.

HCF cells were pretreated with ethanol (vehicle) or 10 μM of Oligomycin A (Sigma-Aldrich, O4876) for 30 min, or with DMSO (vehicle), 5 μM of PP11 or 5 μM of IB13 for 15 min and then loaded with calcein acetoxymethyl ester and Co2+, as previously described [44]. The staining solution was added to the cells for 15 min at 37 °C in a 5% CO_2_ atmosphere. Image acquisitions were performed with a motorized Nikon AX R confocal microscope with a 40×/0.6 PlanApo objective and laser LU-N4S 405/488/561/640. Ionomycin (1 μM) was administered 30 s after the beginning of the experiment to induce PTP opening. Each graph shows the PTP activity expressed as percentage of the slope of each condition, normalized on the slope of control cells (pCMV6 transfected). The data are representative of at least three independent experiments. The graphs show means ± SEM.

### 4.6. Cell Viability Assay (MTS Cell Proliferation Assay)

Cells seeded in 96-well plates were pre-treated with ethanol (vehicle) or 10 μM Oligomycin A for 30 min, or with DMSO (vehicle), 5 μM PP11 or 5 μM IB13 for 15 min. All the treatments were performed at the same concentrations and also for 24 h. Then, MTS reagent (ab197010) was added into each well and incubated for 0.5–4 h at 37 °C in standard culture conditions. Absorbance of treated and untreated cells was measured on a plate reader at OD 490 nm.

### 4.7. Intracellular ATP Level

Total ATP production was measured through an ATP Assay Kit (Abcam, ab83355). In brief, HCF were harvested and pretreated with ethanol (vehicle) or 10 μM of Oligomycin A for 30 min, or with DMSO (vehicle), 5 μM of PP11 or 5 μM of IB13 for 15 min. All the treatments were performed at the same concentration and also for 24 h. The cells were then washed with PBS and resuspended in 100 μL of ATP assay buffer. They were then homogenized and centrifuged (4 °C at 13,000× *g*) to remove any insoluble material. The samples were then treated with Deproteinizing Sample Preparation Kit—TCA (ab204708) according to the manufacturer’s instructions. The supernatants were then collected and incubated with the ATP probe. Absorbance was detected at 570 nm using a microplate reader. The results are presented as percentages, normalized on the vehicle.

### 4.8. Statistical Analysis

One-way ANOVA with multiple comparisons was performed by GraphPad Prism for all experiments. *p* values are reported in the figure legends.

## Data Availability

The data presented in this study are available within the article.

## Supplementary Materials

The following supporting information can be downloaded at: https://www.mdpi.com/article/10.3390/ijms24076191/s1.
