# Supplementary material for: 1,3,8-Triazaspiro[4.5]decane Derivatives Inhibit Permeability Transition Pores through a FO-ATP Synthase c Subunit Glu119-Independent Mechanism That Prevents Oligomycin A-Related Side Effects
Source: Int J Mol Sci. 2023 Mar 24;24(7):6191. doi: 10.3390/ijms24076191 (PMC10094280; doi:10.3390/ijms24076191)
Supplement: Supplementary file 1 [file ijms-24-06191-s001.zip › ijms-2228041-supplementary.pdf]

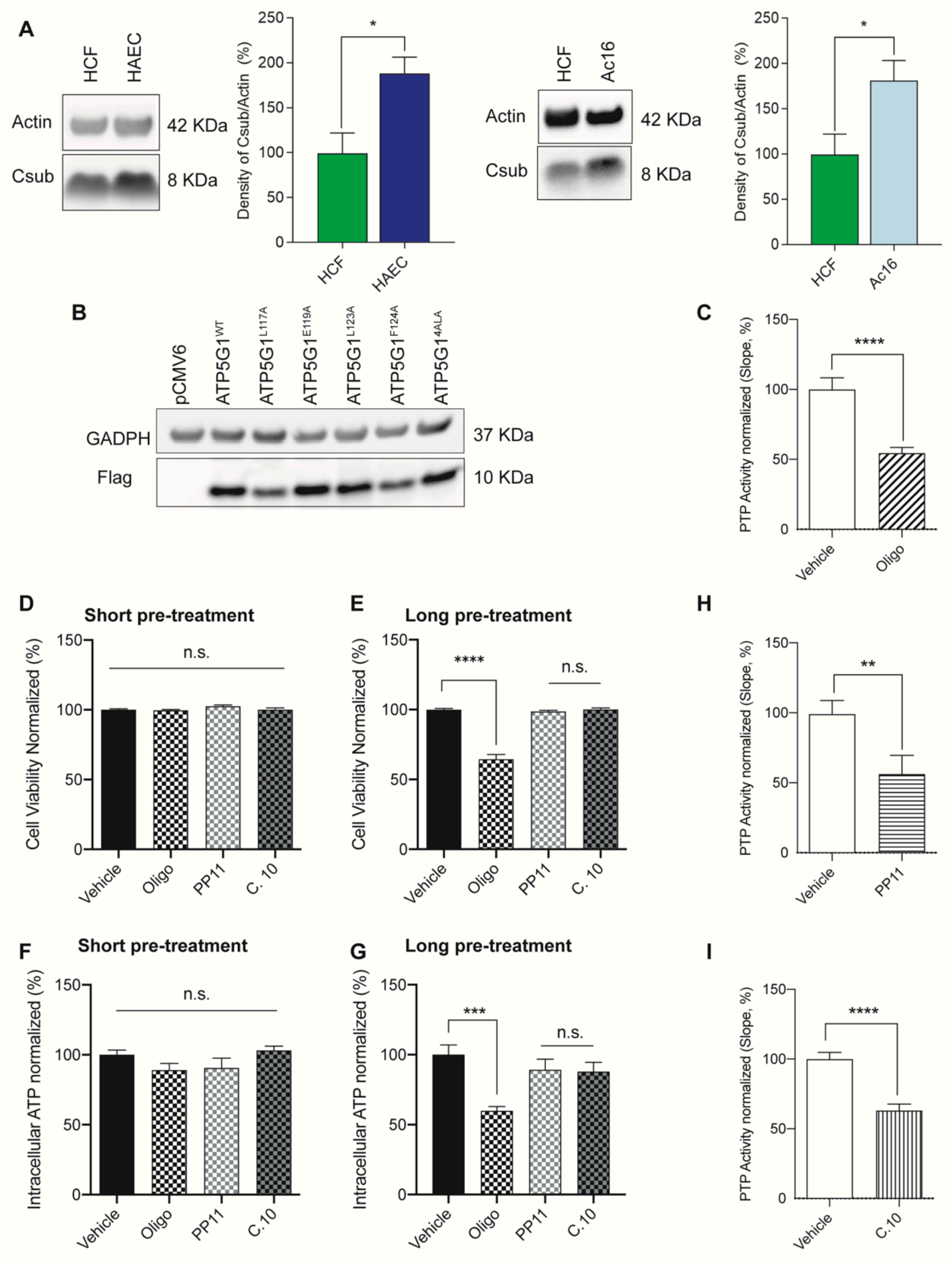

Figure S1. C-subunit protein levels, FLAG-tag protein levels, Cell viability and intracellular ATP levels, PTP activity inhibition with different compounds. (**A**) Representative images of western blot showing Csub protein levels in different cardiac cell lines (HAEC on the left and Ac16 on the right) compared to HCF. Each graph shows the measurement of the signal emitted by Csub protein band normalized on Actin signal in each cardiac cell line. The data are expressed as percentage compared to the Csub/Actin signal of HCF. (**B**) Representative images of western blot showing FLAG-tag protein levels in HCF transfected with the empty vector (pCMV6), wild-type Csub ($ATP5G1^{WT}$) or the Csub mutants. (**C**) PTP activity measurement in non-transfected HCF with vehicle or Oligomycin pre-treatment (10 μM, 30 min). (**D**) Cell viability assay after short pre-treatment with Oligomycin (10 μM, 30 min), PP11 e C.10 (5 μM, 15 min) normalized on vehicle. (**E**) Cell viability assay after long pre-treatment (24 h) with Oligomycin (10 μM), PP11 e C.10 (5 μM) normalized on vehicle. (**F**) Intracellular ATP level after short pre-treatment with Oligomycin (10 μM, 30 min), PP11 e C.10 (5 μM, 15 min) normalized on vehicle. (**G**) Intracellular ATP level after long pre-treatment (24 h) with Oligomycin (10 μM), PP11 e C.10 (5 μM) normalized on vehicle. (**H**) PTP activity measurement in non-transfected HCF with vehicle or PP11 pre-treatment (5 μM, 15 min). (**I**) PTP activity measurement in non-transfected HCF with vehicle or C.10 pre-

treatment (5 μM, 15 min). Statistics differences were analyzed using unpaired t test and one-way ANOVA: **** $p < 0.0001$, *** $p < 0.001$, ** $p < 0.01$, * $p < 0.05$. Data are representative of at least three independent experiments. Graphs show mean ± SEM.
